# Switching between individual and collective motility in B lymphocytes is controlled by cell-matrix adhesion and inter-cellular interactions

**DOI:** 10.1038/s41598-018-24222-4

**Published:** 2018-04-11

**Authors:** Javier Rey-Barroso, Daniel S. Calovi, Maud Combe, Yolla German, Mathieu Moreau, Astrid Canivet, Xiaobo Wang, Clément Sire, Guy Theraulaz, Loïc Dupré

**Affiliations:** 1 INSERM, UMR1043, Centre de Physiopathologie de Toulouse Purpan, Toulouse, France; 2 Université de Toulouse - Paul Sabatier, Toulouse, France; 3 CNRS, UMR5282, Toulouse, France; 4 Centre de Recherches sur la Cognition Animale (CRCA), Centre de Biologie Intégrative (CBI), Centre National de la Recherche Scientifique (CNRS) & Université de Toulouse - Paul Sabatier, Toulouse, France; 5 Laboratoire de Biologie Cellulaire et Moléculaire du Contrôle de la Prolifération (LBCMCP), Centre de Biologie Intégrative (CBI), Centre National de la Recherche Scientifique (CNRS) & Université de Toulouse - Paul Sabatier, Toulouse, France; 6 Laboratoire de Physique Théorique – IRSAMC, Centre National de la Recherche Scientifique (CNRS) & Université de Toulouse - Paul Sabatier, Toulouse, France

## Abstract

Lymphocytes alternate between phases of individual migration across tissues and phases of clustering during activation and function. The range of lymphocyte motility behaviors and the identity of the factors that govern them remain elusive. To explore this point, we here collected unprecedented statistics pertaining to cell displacements, cell:matrix and cell:cell interactions using a model B cell line as well as primary human B lymphocytes. At low cell density, individual B lymphocytes displayed a high heterogeneity in their speed and diffusivity. Beyond this intrinsic variability, B lymphocytes adapted their motility to the composition of extra-cellular matrix, adopting slow persistent walks over collagen IV and quick Brownian walks over fibronectin. At high cell density, collagen IV favored the self-assembly of B lymphocytes into clusters endowed with collective coordination, while fibronectin stimulated individual motility. We show that this behavioral plasticity is controlled by acto-myosin dependent adhesive and Arp2/3-dependent protrusive actin pools, respectively. Our study reveals the adaptive nature of B lymphocyte motility and group dynamics, which are shaped by an interplay between and cell:matrix and cell:cell interactions.

## Introduction

Lymphocytes represent a unique model to study cell motility because they may adopt a vast array of behaviors. These immune cells patrol the organism as isolated individuals endowed with high motility properties favoring the search for pathogens or pathogen-derived determinants^1^. Within tissues, lymphocytes can organize into swarms and clusters to sustain specific steps of activation and function^2–5^. Group organization has also been reported to condition tissue residency of memory lymphocyte populations^6,7^. Furthermore, our recent *in vitro* observations have revealed that the assembly of B and T lymphocytes into clusters confers collective properties leading to distinct chemotactic prowess^7,8^. Understanding the parameters that govern the transition from individual to group behaviors in lymphocytes would provide insight into the mechanisms that determine collective cell behaviors common to many cellular systems. A key question is to disentangle the relative contribution of cell intrinsic versus extrinsic factors in the emergence of collective cell behaviors. To tackle this question, we here reasoned that the characterization of individual cell motility properties and encounter dynamics would explain the emergence of collective organization.

As individual cells, lymphocytes face the challenge of patrolling large tissue areas, while scanning locally to discriminate between cells presenting different antigenic determinants^9^. Lymphocyte migrate as amoeboid cells with patterns characterized as a Brownian random walk^10^, a persistent random walk^11,12^ or a Lévy walk^13^. While both walking behaviors might accommodate local search and tissue diffusion, it is not yet elucidated which parameters might govern their onset^14^. Environmental factors such as extra-cellular matrix (ECM) components affect the motility behavior of lymphocytes^15^. Indeed, lymphocytes can adopt walking versus sliding crawling modes depending on substrate attachment^16,17^. A recent study revealed the presence of “tissue hotspots” that locally influence cell motility properties to favor local search^18^. Furthermore, spatial and temporal changes in ECM composition have been demonstrated to control processes such as lymphocyte recruitment, survival and differentiation^19,20^. Altered expression of fibronectin and collagens I and IV in multiple myeloma is associated to progressive accumulation of malignant lymphocytes in the bone marrow at the different stages of disease progression^21^.

The aim of the present study is to characterize the range of B lymphocyte motility patterns at the single cell level and to decipher which interactions and behavioral mechanisms are involved in the emergence of coordinated groups. Using a well-controlled *in vitro* experimental framework and a dedicated tracking algorithm, we produce unprecedented statistics on B lymphocyte motility properties that highlight both individual heterogeneity and flocking dynamics. Our study reveals that B lymphocyte populations are composed of individuals with distinct diffusion properties that are modulated by interactions with the extracellular matrix. Fibronectin favors B lymphocyte adhesion and increases the proportion of individual cells with high-speed characteristics and chemotactic ability, thereby favoring isolated patrolling. In contrast, reduced adhesion over collagen IV favors group assembly and flocking activity that translate into collective chemotaxis. This study also highlights that actin cytoskeleton remodeling is a key integrator of B lymphocyte adaptive motility properties. Indeed, we identify distinct actin modules that coordinate adhesion to the matrix and protrusive activity involved in cell displacement and encounters with neighbors. Together, our study identifies external cues and intrinsic pathways that explain the wide range of B lymphocyte motility behaviors and control the shift between single versus collective B lymphocyte dynamics.

## Results

### B lymphocytes adapt their adhesion, protrusive activity and motility behavior to matrices

**Figure 1:**
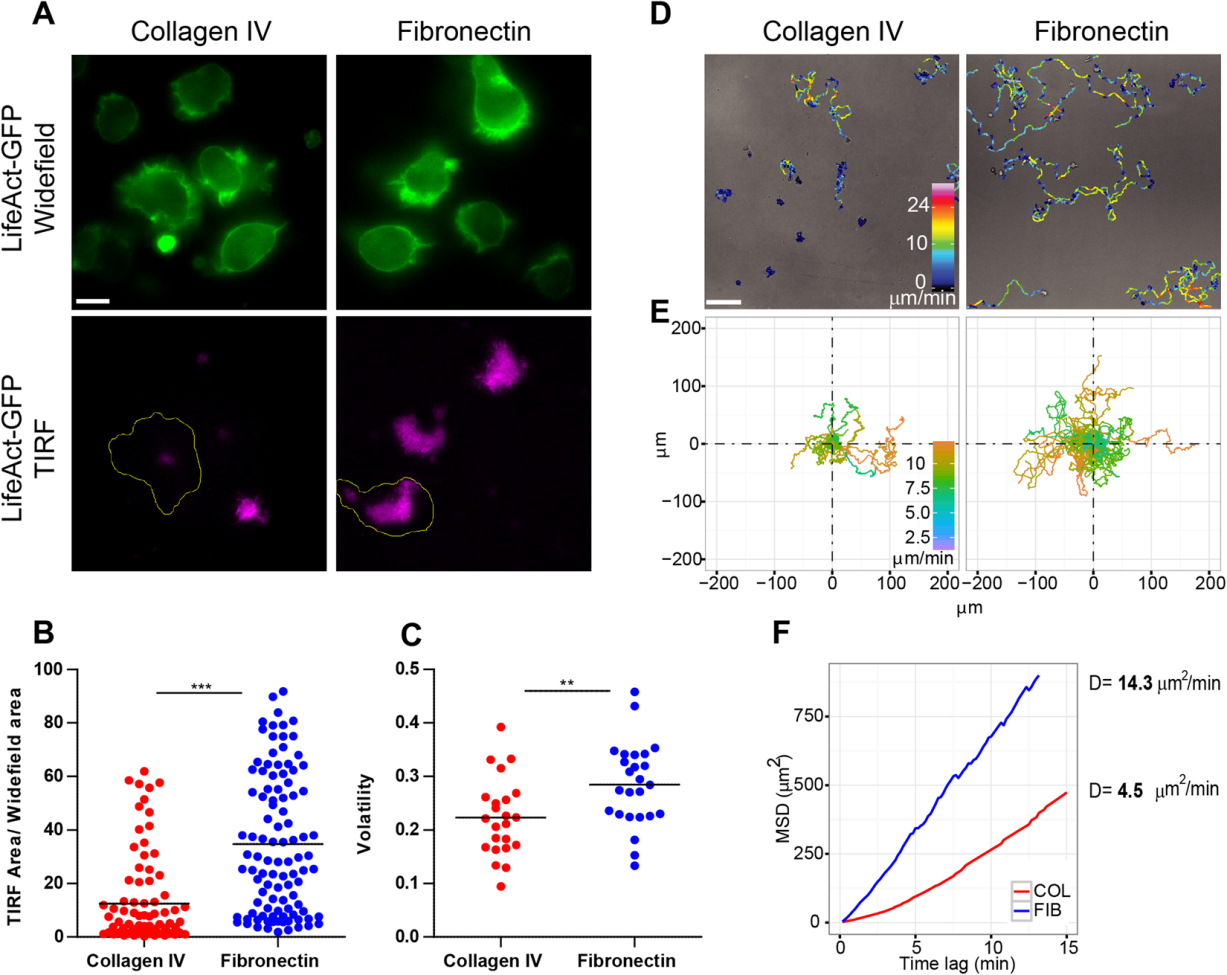
Matrix interaction defines individual motility of B lymphocytes. (**A**) JY cells were seeded at low density (40 cells mm^−2^) over 1,5 μg ml^−1^ collagen IV or 10 μg ml^−1^ fibronectin and imaged after 2 h with combined widefield and TIRF illumination to reveal LifeAct-GFP, respectively throughout the cell body and at the cell/matrix interface. Scale bar, 10 μm. (**B**) The proportion of the surface of LifeAct-GFP detected in the TIRF versus widefield images was measured for each cell (collagen IV, 87 cells; fibronectin, 101 cells; data pooled from 3 independent experiments). Bars show mean values. ***p < 0.001, Student-t test. (**C**) Cell shape volatility represents the standard deviation of each cell aspect ratio changes over consecutive frames (track length, 20 min; time interval, 1 min) over collagen IV and fibronectin (respectively, 23 and 25 cells; data pooled from 3 independent experiments). Bars show mean values. **p < 0.01, Student-t test. (**D**) JY cells were recorded after 2 h of seeding for 12 h at 12 frames min^−1^. Images show representative 1-h tracks with color-coded speed (displacement over 1-min intervals). Scale bar, 100 μm. (**E**) One-hour tracks of 40 cells per condition were normalized to their x,y starting location and color-coded for their speed. (**F**) To quantify diffusion properties, the mean square displacement was plotted as a function of time interval (1-min increment). Data stem from 318 cell tracks on collagen IV and 1700 cell tracks on fibronectin. Diffusion coefficient D was calculated as the slope of the curve. See also Supplementary Figures S1 and S2 and Video S1.

To investigate the influence of cell-matrix interaction on B lymphocyte motility, the human JY B cell line was used as a model system and deposited at low cell density on non-confined 2D surfaces coated with either collagen IV, a main component of the basement membrane with the ability of self-assembly into networks^22^ or fibronectin, a stromal matrix fibrillar component^23^. These ECM components were chosen for their *in vivo* relevance and distinct effects in pathological processes such as wound healing in the spinal cord^20^ and multiple myeloma progression in the bone marrow^21^. The JY cells were checked for the expression of the alpha chains of the integrins known to recognize collagen IV (CD49a, CD49b) and fibronectin (CD49d). Notably, JY cells expressed homogeneous integrin levels, which were comparable to those of primary B cells from the peripheral blood of 3 donors (Supplementary Figure S1). In order to record simultaneously the dynamics of cell adhesion and protrusive activity over the two matrices, we devised a combinatorial approach including interference reflection microscopy (IRM), total internal reflection fluorescence (TIRF) imaging of actin dynamics at the contact with the matrix, and wide-field illumination to monitor whole-cell actin remodeling (Supplementary Figure S2). TIRF imaging of LifeAct-GFP (purple) reveals a dense and dynamical actin network that co-localized with the dark areas on parallel IRM snapshots, confirming that it corresponds to the matrix contact area (Supplementary Figure S2). On the other hand, wide-field recording of LifeAct-GFP (green) shows actin enrichment at the cell cortex and within protrusions, which are emitted above the TIRF plane. This combinatorial approach therefore suggests a segregation of the actin cytoskeleton into an adhesive and a protrusive module. This approach indicates that B lymphocytes engage in higher attachment with fibronectin than collagen IV, while they emit various protrusions over both matrices (Fig. 1A). Cell attachment was quantified as a ratio between the TIRF area (adhesive actin) and the cell contour (protrusive actin) (Fig. 1B). This analysis reveals that collagen IV and fibronectin promote very distinct cell attachment, as assessed by the adhesive area. These distinct behaviors are also observed in peripheral blood primary B cells (Supplementary Figure S3A,B).

We then asked how matrix composition would affect B lymphocyte protrusive activity. For that purpose LifeAct-GFP expressing cells were imaged over time as they interacted with collagen IV or fibronectin (Supplementary Video S1) and their shape volatility (Fig. 1C), defined as the standard deviation of the aspect ratio changes over consecutive frames^24^ was measured. This analysis reveals that cells over fibronectin remodel their shape more dynamically than over collagen IV. We then assessed whether the adhesive and protrusive activities of B lymphocytes over fibronectin and collagen IV would be translated into distinct motility behaviors. For that purpose, the motility of individual B lymphocytes was recorded at high temporal resolution (5-sec time-lapse) and over prolonged time periods (up to 12 h) using bright-field illumination to avoid phototoxicity. A dedicated algorithm based on local light contrast changes around the cell perimeter was designed to automatically recognize and track hundreds of cells on different video-recordings yielding robust motility statistics (see Methods). B lymphocytes display distinct motility behaviors over the two matrices (Supplementary Video S1). First, the speed (indicated by the color code along the trajectories) of B lymphocytes is relatively low over collagen IV (0 to 10 μm min^−1^ range for most of the cells), while it alternates between slow and high values for most of the cells over fibronectin (0 to 20 μm min^−1^ range) (Fig. 1D). Such values are in agreement with the velocities measured for primary lymphocytes *in vitro* and *in vivo*^13,14,24,25^. Second, analysis of 1-h displacements (Fig. 1E) shows a further distance covered when B lymphocytes were deposited on fibronectin, as compared to collagen IV. Furthermore, analysis of mean square displacement (MSD) over time (Fig. 1F) shows that for both matrices the diffusion exponent is close to 1 indicating that independently from matrix composition, B lymphocytes move as normal diffusers. However, B lymphocytes adopt distinct diffusive characteristics over the two matrices since they move with a diffusion coefficient of 4.5 μm^2^ min^−1^ over collagen IV and of 14.3 μm^2^ min^−1^ over fibronectin. Notably, the higher cell motility over fibronectin as compared to collagen IV also applies to primary B cells (Supplementary Figure S3C). These data highlight that B lymphocytes adapt their motile behavior to matrix composition by modulating both their actin-rich adhesive surface and cell shape dynamics.

### B lymphocyte motility at the population and individual cell scales

**Figure 2:**
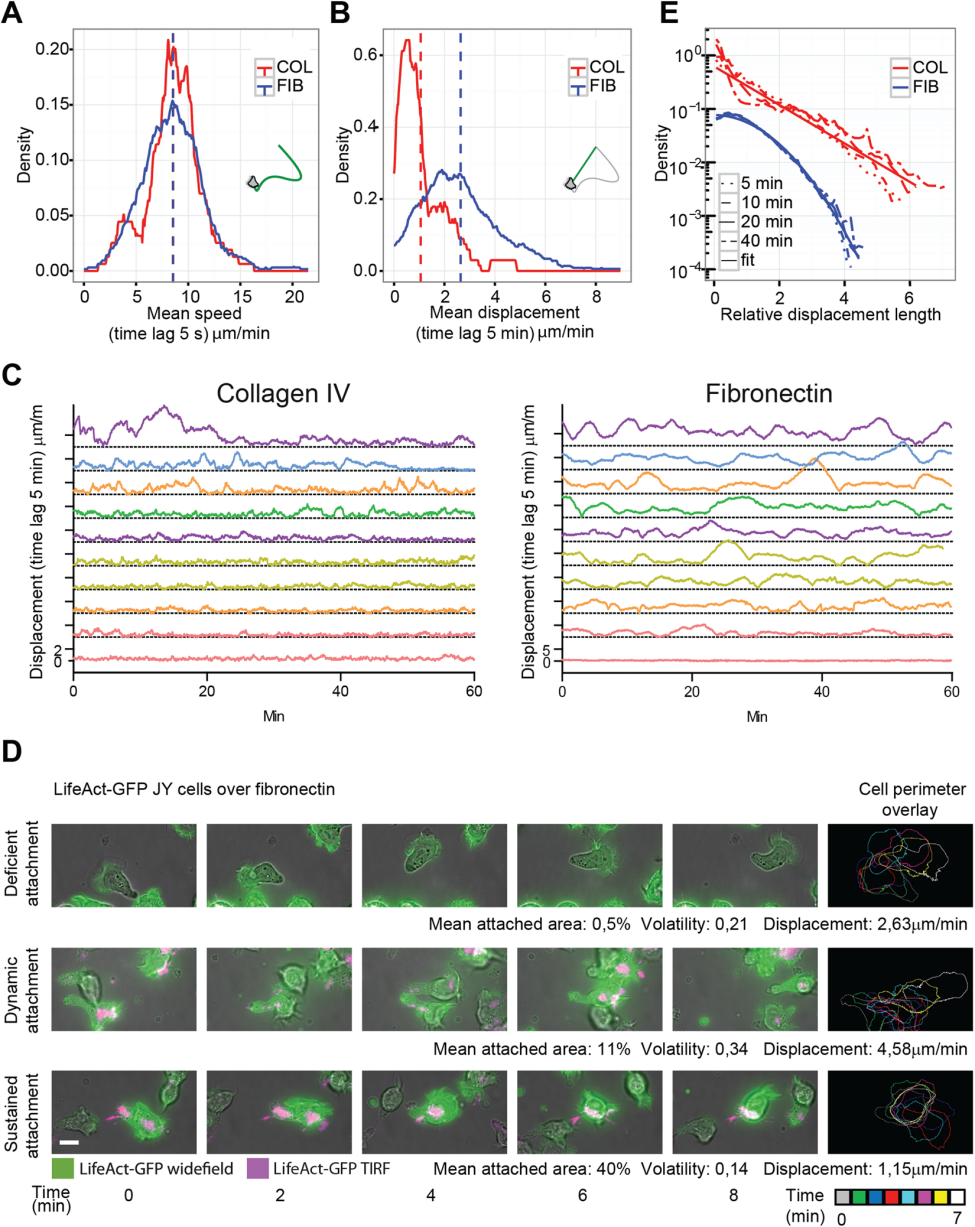
B lymphocytes adopt distinct walking behaviors depending on matrix composition. (**A**) Mean speed of JY cells migrating for at least 1 h over collagen IV or fibronectin was calculated as full accumulated distance (tracks with 5-sec time resolution) divided by track time length (collagen IV, 90 cells; fibronectin, 330 cells; data from 10 experiments). Dashed bars represent mean values. (**B**) Mean displacement from the same cell tracks was calculated as the mean of euclidean distances over 5-min time lags. Dashed bars represent mean values. (**C**) Time evolution of 5-min cell displacements for 10 representative JY cell tracks over each matrix. (**D**) Representative lifeact-GFP-expressing JY cells migrating over fibronectin and imaged for 10 min with a combination of brightfield, widefield and TIRF modes. Panels to the right represent a color-coded overlay of cell shape and position over 2-min intervals. Mean attached area, volatility and displacement were calculated as described above. Scale bar, 10 μm. (**E**) Cell displacements over collagen IV or fibronectin were computed using a time lag of 5, 10, 20 and 40 min and normalized to their mean length. Data stem from 63 cell tracks on collagen IV and 229 cell tracks on fibronectin. For readability, the fibronectin curve was shifted downwards by a factor 8. See also Supplementary Video S2.

To further investigate which motility parameters might explain the distinct diffusive characteristics of B lymphocytes over the 2 model matrices, we measured, for each cell, the mean instantaneous speed (time resolution of 5 sec) and the mean displacement over 5-min periods. Our analysis reveals that the distribution of the mean instantaneous speeds of individual B lymphocytes is very similar over the 2 matrices (Fig. 2A), while the average displacement of the cells is three times higher over fibronectin than collagen IV (Fig. 2B). These results suggest that the higher diffusive property of B lymphocytes on fibronectin is not linked to a distinct local scanning activity, but to the ability to translate local movements into more effective exploratory cell displacements. The average displacement over both matrices is distributed over a wide range of values. This might result from a variable behavior of individual cells over time or from stable but heterogeneous properties at the individual cell level. To address this point, the evolution of the average displacement was followed for individual cells over 1 h. This analysis reveals that B lymphocyte motility is characterized by numerous acceleration/deceleration cycles over both matrices and that individual cells undergo these speed variations within a characteristic speed range (Fig. 2C). We then sought to explore whether specific morphological attributes might be associated to the widely distributed diffusive properties. For that purpose, we used our combinatorial microscopy approach to track cell-matrix interaction in cells displaying different diffusion abilities (Fig. 2D; Supplementary Video S2). Cells that lack a detectable actin-rich matrix attachment, although displaying high protrusive dynamics, have modest diffusion abilities. Cells that distribute adhesive structures into dynamic patches aligned along the cell polarity axis are displaying high displacement. This is reminiscent of the walking behavior described previously in T lymphocytes^17^. Cells that display large and sustained matrix attachment fail to displace efficiently, probably because of a reduced ability to detach from the matrix. Together, these observations indicate that the heterogeneity of B lymphocytes diffusive properties is mirrored by distinct actin-rich patterns of matrix attachment.

To further characterize the diffusion properties of B lymphocytes over the two model matrices, we analyzed the distribution of individual displacement lengths normalized by their mean value, for time lags of 5, 10, 20, 40 minutes (Fig. 2E). In both cases, the different densities collapse on the same curve after this normalization. However, while over fibronectin the distribution is nearly Gaussian, like for usual Brownian walkers, displacements over collagen IV display a much wider distribution. These results therefore demonstrate that B lymphocytes adopt different diffusive properties depending on matrix composition, moving as fast Brownian-like random walkers over fibronectin and as slow non-Gaussian walkers over collagen IV, with a much wider range of displacements.

### B lymphocyte adhesion to matrix counteracts their assembly into clusters

**Figure 3:**
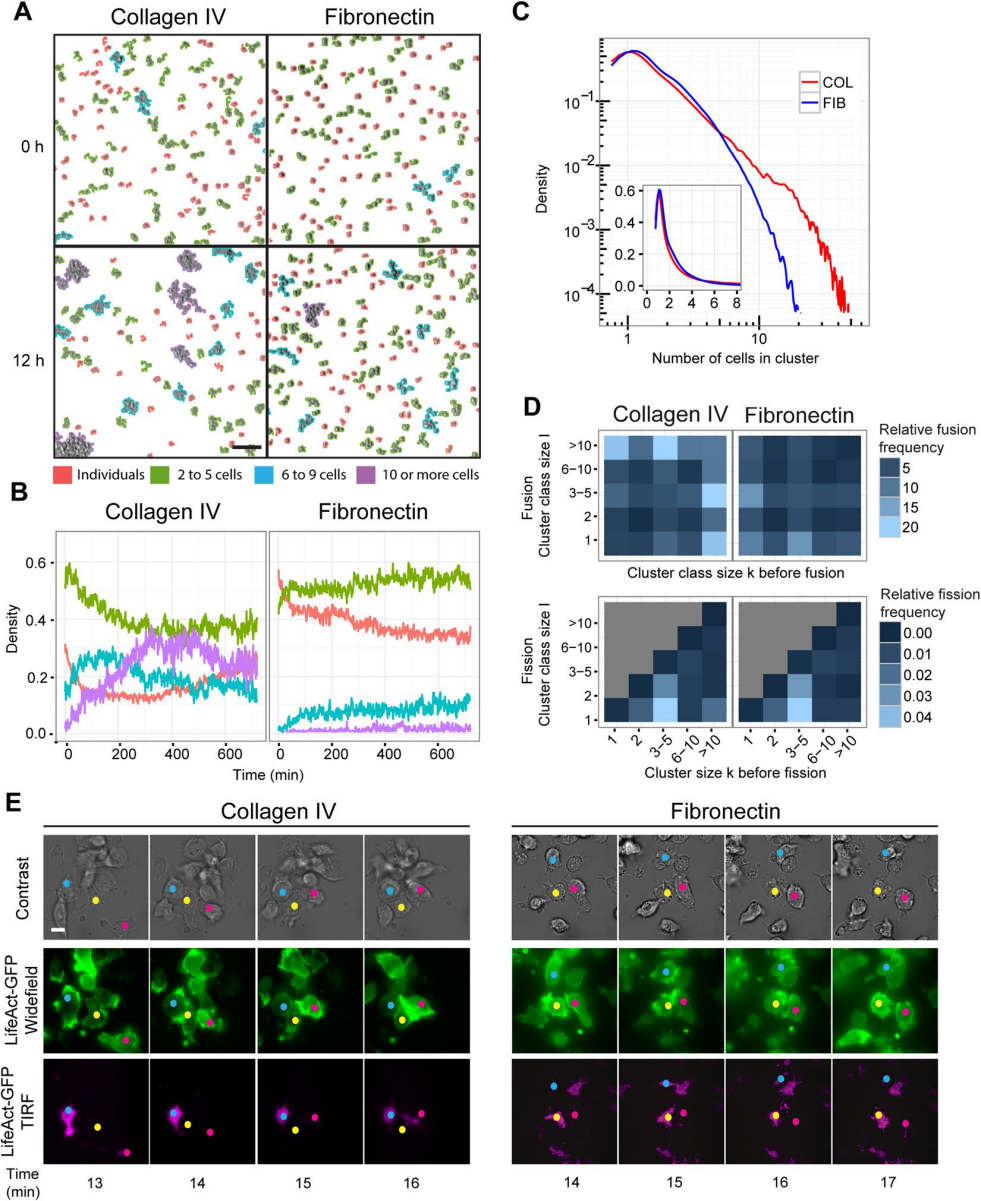
Extracellular matrix conditions assembly of B lymphocytes into clusters. (**A**) JY cells were seeded at high cell density (1000 cells mm^−2^) over collagen IV or fibronectin and recorded for 12 h. Individual cells and cell clusters were color-coded according to the following size categories: individual cells, 2–5 cells, 6–9 and ≥10 cells. Representative images at time points 0 and 12 h are shown. Mean cell number: 1831 over Collagen IV and 1788 over fibronectin. Scale bar, 100 μm. (**B**) Temporal evolution of the relative area covered by each size category. Data were extracted from 10 videos per condition. (**C**) Relative distribution of clusters of increasing size studied at the stationary phase (6–12-h time window). (**D**) Relative frequency of fusion and fission events between clusters of different sizes occurring during the 6–12-h time window. See also Supplementary Figure S2 and Video S3. (**E**) Representative images of JY cells expressing Lifeact-GFP seeded over collagen IV or fibronectin and recorded over 10 min with a combination of brightfield, widefield and TIRF modes to reveal actin dynamics in cell encounters. Colored dots were added onto cells to keep track of their identity in the successive snap shots. Scale bar, 10 μm. See also Supplementary Video S4.

Although lymphocytes are generally considered as solitary cells, they face multiple physio-pathological settings at high cell density that favor homotypic contacts. It is currently unknown how cell-matrix and cell-cell interactions are integrated to control lymphocyte motility. To address this question, B lymphocytes were seeded at high density (1000 cells mm^−2^) over collagen IV or fibronectin and followed for 12 h with our tracking algorithm, for which a cluster size color-coded classifier and a fusion/fission counter were implemented (Supplementary Video S3). Over collagen IV, B lymphocytes assemble into cell clusters, whose size increases progressively over approximately 6 h, resulting in a large proportion of cells being associated to large-size clusters (Fig. 3A,B). The video recordings also reveal a dynamic exchange of cells between the different categories of clusters’ sizes. In comparison, cell aggregation over fibronectin is reduced. Indeed, even if the proportion of isolated cells progressively declines over time, a smaller number of large clusters emerge. This is confirmed by the analysis of the cluster size distribution at the stationary phase (>6 h), which decays much faster over fibronectin than over collagen IV (Fig. 3C). Similarly, interaction with collagen IV favors primary B cells aggregation, while fibronectin tends to limit it (Supplementary Figure S3D,E). To disentangle whether the distinct cellular behaviors observed over the 2 model matrices were related to the nature of the matrix, rather than its coated concentration, we further studied the B cell clustering process using low (1.5 µg/ml) and high (10 µg/ml) concentrations of fibronectin and collagen IV, as well as a non-coated surface (Supplementary Figure S4). Our data clearly support the notion that collagen IV favors assembly of large clusters and that this process is reinforced at high concentration (10 µg/ml). In contrast, fibronectin prevents the clustering behavior at both tested concentrations. These findings indicate that the nature of fibronectin and collagen IV, rather than their local concentrations have diametrically opposite effects on B lymphocyte aggregation. The increased degree of B lymphocyte aggregation observed over collagen IV might result from distinct dynamics of interactions between cells. Indeed, the quantification of fusion probabilities as a function of cluster size reveals that collagen IV favors the fusion of individual cells or small groups together with large groups (>10 cells), while fusion events over fibronectin are mostly limited to individual or small clusters among themselves (Fig. 3D). On the other hand, fission probabilities are comparable over the two matrices, with most fission occurring in small-size clusters (3–5 cells) (Fig. 3D). This implies that interaction with fibronectin hampers B lymphocyte aggregation by decreasing fusion events leading to the assembly of large groups. These data suggest that distinct cell-matrix interactions influence cell-cell interaction dynamics leading to cluster assembly. Such interplay might be controlled via actin cytoskeleton remodeling as recently investigated in the *Dictyostelium* model^26^. To explore this possibility in our model, LifeAct-GFP transfected JY cells were imaged at high cell density over collagen IV or fibronectin using our combinatorial microscopy approach (Fig. 3E; Supplementary Video S4). Most cells over collagen IV establish limited and unstable actin-rich attachment areas, while they emit actin-rich pseudopodia and lamellipodia in upper planes. The collisions between cells initially occur via the actin-rich protrusions, which then retract and reorient away from the inter-cellular contact. This behavior favors the juxtaposition of cell uropods, which appear to be the preferential structure through which cells engage into stable adhesions. This stepwise behavior results in the generation of clusters made of radially oriented cells, as previously observed for leukemic cells^7^ and neural crest cells^27^. Over fibronectin, a higher proportion of cells engage in sustained attachment to the substrate. When colliding, cells either reorient their migration or slide along each other without stabilizing inter-cellular contacts. In contrast to the behavior observed over collagen IV, neighbor cells do not appear to attach via their uropods, which are instead strongly engaged into matrix attachment, as revealed by the filamentous structures observed in the TIRF plane. A combination of contact-induced reorientation, sliding and uropod attachment to the matrix therefore contributes to prevent the generation of stable clusters over fibronectin. Together, these data suggest that the actin cytoskeleton integrates the interactions of cells with extracellular matrix and other cells to differentially control cell decision to assemble into groups or remain isolated.

### Actin branching and actomyosin contraction control the balance between individual and collective behaviors

**Figure 4:**
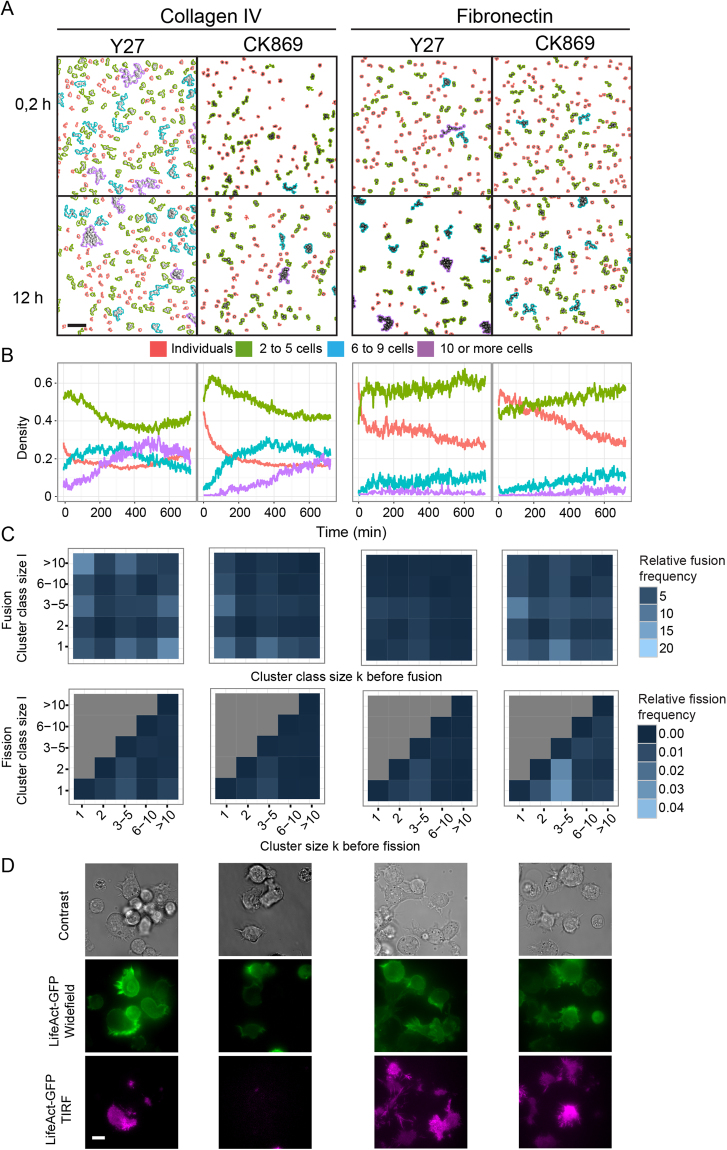
B lymphocyte motility and clustering are differentially controlled by actin branching and actomyosin contraction. (**A**) JY cells pretreated with Y27632 or CK869 were seeded at high cell density over collagen IV or fibronectin and recorded for 12 h. Representative images showing color-coded cells and clusters according to their size are shown at time points 0.5 and 12 h. Scale bar, 100 μm. (**B**) Temporal evolution of the relative area covered by each size category. Data were extracted from 10 videos per condition (mean cell number: 1831 over Collagen IV and 1788 over fibronectin). (**C**) Distribution of relative fusion and fission frequencies according to cell cluster size. Data were extracted from the stationary phase (6–12-h time window). Number of fusions considered: 109549 on collagen IV and 170200 on fibronectin, number of fissions considered: 10737 on collagen IV and 10949 on fibronectin. (**D**) Representative images of JY cells expressing Lifeact-GFP seeded over collagen IV or fibronectin and imaged with a combination of brightfield, widefield and TIRF modes to reveal actin dynamics in cell encounters. Scale bar, 10 μm. See also Supplementary Figure S3 and Video S5.

To investigate how the identified actin cytoskeleton processes might control emergence of B lymphocyte collective behaviors, we used chemical inhibitors to specifically block ROCK1-dependent actomyosin contractility (Y27632) and Arp2/3-dependent actin branching (CK869). Over collagen IV, both the blockade of ROCK1 and Arp2/3 reduce cell diffusion (Supplementary Figure S5A), as reported in previous studies^28–31^. While ROCK1 blockade appears to affect cell motility by enhancing cell adherence (Supplementary Figure S5B), Arp2/3 blockade does not alter cell adherence but affects the stability of protrusions. ROCK1 inhibition delays the emergence of large clusters over collagen IV (Fig. 4A,B *vs* Fig. 3A,B). This is mirrored by reduced frequency of both fusion and fission events (Fig. 4C *vs* Fig. 3D), which reflects the reduced diffusive activity of individual cells, mostly due to a decrease in their mean speed (Supplementary Figure S3). Arp2/3 blockade has an even stronger effect on the aggregation of B lymphocytes over collagen IV, with a marked defect in the assembly of large clusters (Fig. 4A,B *vs* Fig. 3A,B). The global reduction of fusion events (Fig. 4C *vs* Fig. 3D) can be explained by the reduction of B lymphocyte diffusion, caused not only by a speed reduction but also by a decreased ability to translate it into active displacement (Supplementary Figure S5C). CK869 treatment induced the near disappearance of fusions involving medium- to large-size clusters, revealing a qualitative change in cell-cell interactions. We further used our combinatorial microscopy approach to investigate how the blockade of ROCK1 and Arp2/3 might affect the cell-matrix and cell-cell interaction dynamics. While ROCK1 inhibition enhances cell-matrix attachment, it does not appear to affect the ability of cells to engage contacts with neighbors (Fig. 4D; Supplementary Video S5). As previously reported, cells inhibited for Arp2/3 activity display erratic protrusive activity^32^. Furthermore, treated cells display a reduced ability to stabilize contacts upon encounter, which indicates that the quality of cell protrusions is key to the assembly of B lymphocytes into clusters.

We then investigated how the blockade of ROCK1 and Arp2/3 might modulate the dynamics of cell encounters over fibronectin. Both treatments reduce B lymphocyte diffusion (Supplementary Figure S3). While ROCK1 inhibition mainly affects cell main speed, Arp2/3 inhibition reduces both speed and displacement ability (Supplementary Figure S3). ROCK1 blockade enhances cell adherence, while Arp2/3 blockade decreases it (Fig. 4B). Over fibronectin, which does not promote B lymphocyte aggregation, the blockade of ROCK1 and Arp2/3 has no noticeable impact on clustering, nor on the distribution of fusions and fissions events. At high cell density, ROCK1 blockade appears to impede cell detachment from fibronectin and it does not appear to affect the ability of cells to engage contacts with neighbors (Fig. 4D; Supplementary Video S5). Arp2/3 blockade promotes erratic protrusion dynamics that does not influence the limited ability of cells to aggregate over fibronectin.

These results highlight that actin cytoskeleton dynamics coordinate the interactions of B lymphocytes with matrix and neighbor cells. In particular, Arp2/3-dependent actin branching plays a key role in stabilizing contacts between cells and cluster assembly. On the other hand, actomyosin contraction sustains cell migration by regulating cell-matrix interaction dynamics, while it is dispensable for cluster assembly.

### Onset of B lymphocyte collective motility depends on matrix composition

**Figure 5:**
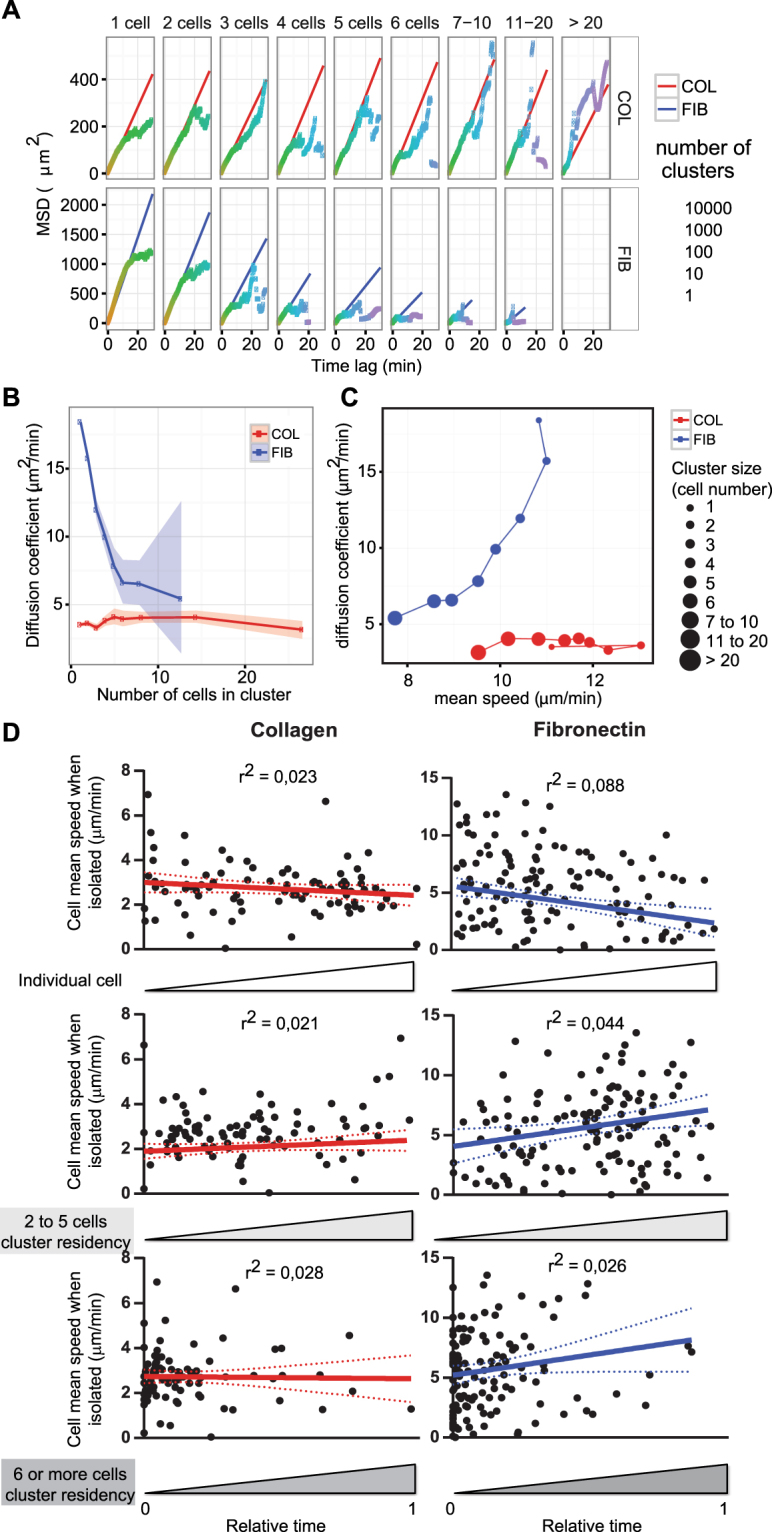
Emergence of B lymphocyte collective motion properties depends on matrix interaction. (**A**) Mean square displacement was plotted as a function of time interval for JY cell clusters of growing size tracked at high cell density over collagen IV and fibronectin. Linear regressions and shown in red or blue, respectively. The color code indicates the number of tracks available for each time length. Data were pooled from 10 videos per condition. (**B**) Diffusion coefficient of each cluster category represented with confidence interval. (**C**) Correlation between diffusion coefficient and mean speed (time lag of 5 s) is shown for clusters of each category. (**D**) JY-GFP cells diluted 1/40 with unstained JY cells, seeded at high cell density over collagen IV or fibronectin and imaged after 6 h for another 6 h, each 30 s. GFP cells were automatically tracked and their time as isolated cells or as part of clusters were quantified. The plots represent the mean speed of each cell as it migrated alone, as a function to the relative time of those cells as isolated cells, as part of small clusters or as part of large clusters. Linear regression and 95% confidence interval were plotted (collagen IV, 90 cells; fibronectin, 132 cells; data from one representative experiment out of 3). See also Supplementary Figure S4 and Video S6.

Collective behaviors in biological systems emerge from a complex set of interactions between individual components that are modulated by external conditions in the environment^33–35^. With the aim of understanding if the interactions of B lymphocytes with the matrix modulate not only aggregation, but also the possible onset of collective motion, individual cell and cluster motility was analyzed on fibronectin and collagen IV. Groups of increasing size were tracked on both matrices to calculate the MSD over time (Fig. 5A) and the diffusion coefficient (Fig. 5B). The data show that over fibronectin diffusion decreases with cluster size, as expected for non-cooperative walkers. In contrast, clusters formed over collagen IV display a sustained diffusive ability, which remains relatively constant whatever the group size. This suggests that clusters over collagen IV might be endowed with a diffusive advantage. Matrix influence on cluster diffusion properties was further studied by analyzing the relationship between diffusion speed and average speed for various cluster sizes (Fig. 5C). Over fibronectin, both motile parameters decay as a function of group size, partially explaining the diffusive defect of the groups by a diminution of their speed. Over collagen IV, the maintenance of diffusion coefficient is independent from changes in average speed. The diffusive property of B lymphocyte clusters over collagen IV might result either from the emergence of a cooperative behavior between cells or from a biased assembly of the most motile B lymphocytes into clusters. To discriminate between these two possibilities, GFP-expressing cells were mixed with unstained ones at a 1/40 ratio in order to track their motility and calculate the time they spent as isolated cells or as members of clusters (Fig. 5D; Supplementary Figure S4; Supplementary Video S6). Over fibronectin, the average displacement of the cells weakly and inversely correlates with the time they remained isolated. This is most probably caused by an increased probability of the most rapid cells to encounter neighbor cells. On the contrary, over collagen IV, cell propensity to remain isolated or to assemble into clusters is independent from their average displacement. This suggests that the sustained diffusion of clusters formed over collagen IV does not result from the aggregation of the fastest cells but arises from a cooperation mechanism between cells. To address whether clustering would affect the motility properties of individual cells, we tracked the stained cells and compared their motility as isolated cells or as part of clusters (Supplementary Figure S4). Over collagen IV, the speed of individual cells increases as they join clusters, indicating that assembly into clusters confers superior motility properties to the cells. Distinctly, over fibronectin, the speed of the cells is similar as they migrate as isolated individuals or as part of clusters. This is in apparent contrast to the loss of diffusive properties of the clusters (Fig. 5B). A possible interpretation is that, over fibronectin, cells remain motile in clusters but slow down cluster displacement because of a lack of coordination. Together, these data indicate that interactions of cells with the matrix condition the emergence of coordination leading to collective migration.

### ROCK1 activity plays a pivotal role in the control of B lymphocyte collective chemotaxis

**Figure 6:**
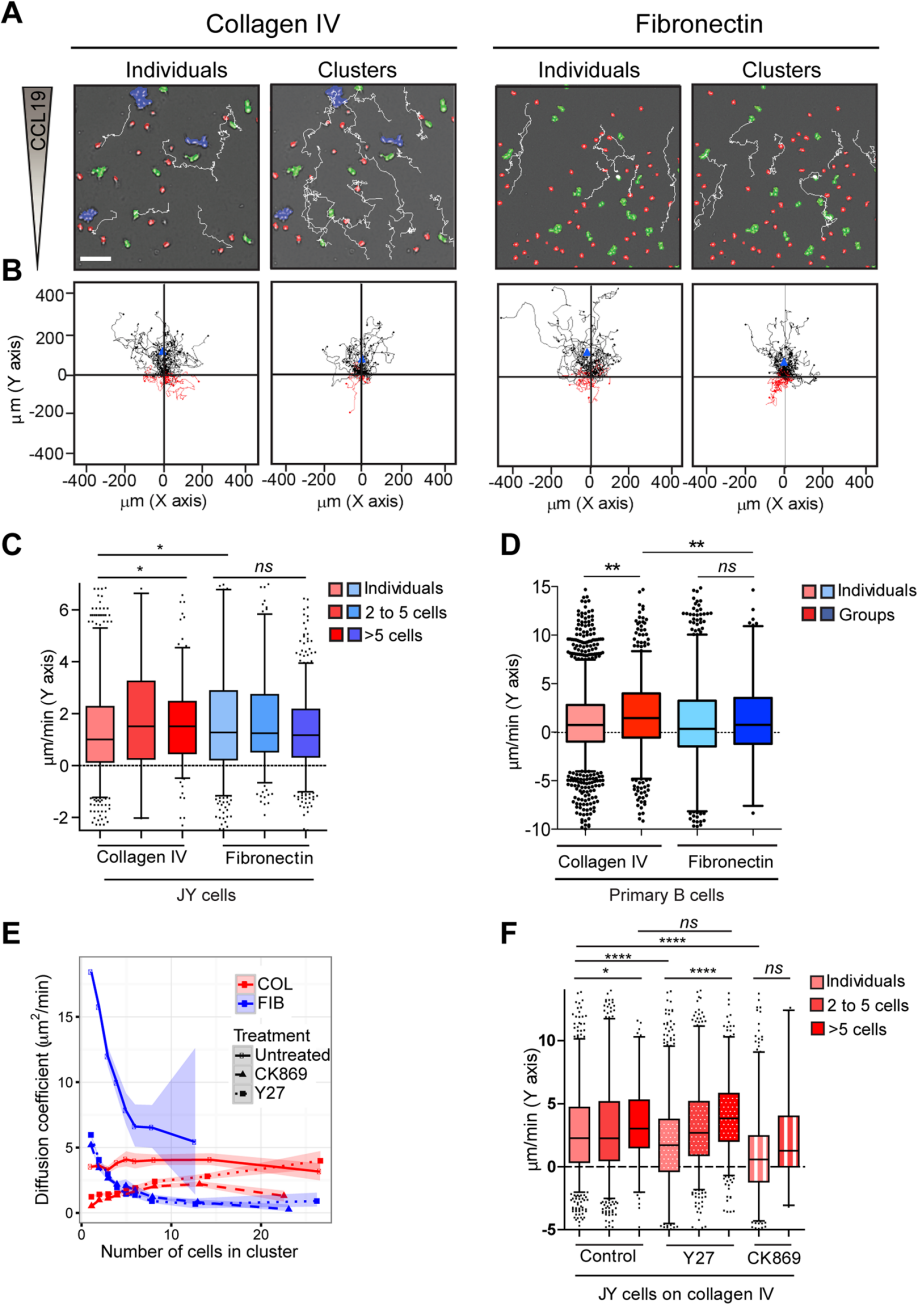
Collective chemotaxis of B lymphocytes depends on matrix interaction and is differentially controlled by actin branching and actomyosin contraction. (**A**) JY cells migrating over collagen IV or fibronectin in Ibidi chemotaxis chambers in which a 0-500 ng ml^−1^ CCL19 gradient was established. Colors represent the following size categories: individual cells (red), 2–5 cell clusters (green) and ≥6 cell clusters (blue). Representative tracks of individual cells and cell clusters are shown. Scale bar, 100 μm. (**B**) One-hour tracks of 40 cells or clusters per condition were normalized to their x,y starting location. Blue triangles mark the population center of mass at 1 h. Black and red tracks indicate displacement over 1 h, respectively towards and away from the chemokine source. (**C**) Chemotactic speed calculated as the mean displacement per min along the gradient axis is shown for each size category, with a 5 to 95% data range and values out of the range as single points. Data were pooled from 3 experiments, yielding 249 to 921 values, depending on the size group and matrix. *p < 0.05, ANOVA test with Sidak-Holms correction for multiple pairs-comparisons. (**D**) Primary B cells were seeded over collagen IV or fibronectin, exposed to a 0–100 ng ml^−1^ CCL19 gradient and tracked for 12 h. Chemotactic speed is shown for each size category, with a 5 to 95% data range and values out of the range as single points. Data correspond to the pool of 2 experiments, including 213 to 2192 values, depending on the size group and matrix. **p < 0.01, ANOVA test with Sidak-Holms correction for multiple pairs-comparisons. See also Supplementary Figure S4 and Video S7. (**E**) JY cells were treated with Y27632 or CK869 and tracked over collagen IV or fibronectin. Diffusion coefficients of the indicated size categories are represented with confidence intervals. For comparison, diffusion from untreated cells and clusters is also represented. (**F**) JY cells were treated with Y27632, CK869 or 0,1% DMSO, seeded over collagen IV and exposed to a 0–500 ng ml^−1^ CCL19 gradient. Chemotactic speed is shown for each size category, with a 5 to 95% data range and values out of the range as single points. Data were pooled from 2 experiments, yielding 137 to 1924 values, depending on the size group and matrix. *p < 0.05, ****p < 0.0001, ANOVA test with Sidak-Holms correction for multiple pairs-comparisons. See also Supplementary Figure S4 and Video S8.

We previously established that malignant lymphocyte clusters display increased chemotactic ability as compared to individual cells^7^. We here tested how B lymphocyte interaction with different matrices might modulate the emergence of chemokine-evoked collective directional motility. For that purpose, JY cells were exposed to a CCL19 chemokine gradient set in dedicated chemotaxis chambers coated with either collagen IV or fibronectin (Fig. 6A; Supplementary Video S7). Isolated cell and cluster trajectories were tracked automatically (Fig. 6B) and the displacement towards the chemokine was computed (Fig. 6C). Over collagen IV, B lymphocyte clusters reach further distances in the direction of the chemokine source as compared to isolated cells. This illustrates how the coordination between cells gives rise to increased chemotactic properties. Over fibronectin, the displacement towards CCL19 of isolated B lymphocytes was higher than that of their counterparts over collagen IV. This is consistent with the fact that B lymphocyte adherence over fibronectin enhances their basal motility, which might translate into enhanced chemotaxis. In contrast to collagen IV, fibronectin does not promote collective chemotaxis. Indeed, B lymphocyte groups display reduced displacement towards CLL19, as their size increases. Our interpretation of these data is that the extended cell interaction with fibronectin favors individual cell chemotaxis, while preventing cell coordination required for collective chemotaxis. Primary B cells also undergo a higher chemotactic speed over fibronectin than over collagen IV (Fig. 6D). Importantly, primary B cells are also capable of collective chemotaxis since clusters over collagen IV display increased chemotactic speed as compared to isolated cells. Differently, over fibronectin, primary B cell clusters do not display chemotactic advantage over individual cells.

Our experimental model shows that both diffusion coefficient and chemotactic speed decrease with group size over fibronectin, whereas they both increase over collagen IV. This is reminiscent of the fluctuation-dissipation theorem derived from the Einstein relation^36^, which states that for physical particles, the diffusion coefficient is proportional to the response to the applied field (Supplementary Figure S4).

We then explored which aspects of actin cytoskeleton remodeling might contribute to the emergence of B lymphocyte collective motility. Groups of increasing size were tracked on both matrices to calculate the MSD over time (Supplementary Figure S4) and the diffusion coefficient (Fig. 6E). The data show that over fibronectin, although the actin blocking treatments alter cluster diffusion, the reduction of diffusion as a function of size is maintained. Over collagen IV, Arp2/3 blockade reduces diffusion independently from cluster size. Interestingly, although ROCK1 blockade affects the diffusion of individual cells and small clusters, it spares that of large clusters. This result, combined to the observation that ROCK1 blockade reduces cell cluster speed (Supplementary Figure S4), indicates that this treatment promotes effective displacement of clusters, possibly by reinforcing a cell-to-cell cooperative process.

We further studied how the differential effect of ROCK1 blockade on the diffusion of individual cells and clusters in the absence of gradient would translate in the context of a chemokine field. While Y27632 treatment results in a reduction of individual cell chemotactic ability, it enhances the chemotactic speed of large clusters (Fig. 6F; Supplementary Video S8). Differently, CK869 treatment affects both the chemotaxis speed of individual cells and of the few stable clusters that are formed under this condition. Together, our study reveals that ROCK1-controlled actomyosin contractility plays a pivotal role in the adaptive motility behavior of B lymphocytes by tuning differentially individual cell motility and collective chemotaxis.

## Discussion

Our study reveals a wide range of B lymphocyte motility behaviors that emerge from the interplay between cell-matrix and cell-cell interactions. In particular, interactions of cells with the extracellular matrix determines not only their motility behavior as individual cells, but also their propensity to assemble into homotypic clusters and to undergo collective chemotaxis. Our study further highlights the key role played by apparently distinct actin cytoskeleton compartments to integrate the effects of cell-matrix and cell-cell interactions to control the onset of collective dynamics in B lymphocyte populations.

A first key finding of our study is that B lymphocytes adapt their motility behavior to their environment. Indeed, depending on matrix composition, B lymphocytes are either standard Gaussian random walkers or persistent random walkers characterized by a wide distribution of displacements. The adaptive property of lymphocytes could reconcile the different motility behaviors assigned to lymphocytes in different studies^10,13,14^. Interestingly, lymphocytes have been suggested to adopt either Brownian random walks or persistent random walks within non-inflamed lymph nodes^14^. In light of our data, this apparent dichotomy might result from the adaptability of lymphocyte populations in response to distinct ECM components composing the lymph node^17,37^. Beyond the motility behaviors identified with our simplified *in vitro* assays, lymphocytes may adopt additional motility behaviors when facing more complex environments^1,38,39^. As lymphocytes extravasate, they migrate successively through ECM environments made of distinct molecular compositions and architectures, such as collagen IV-containing basement membranes and fibronectin-rich fibrillar matrices^40^. Although combinations of matrix components were not assessed in our work, our data suggest that lymphocytes may adapt their motility behaviors as they shift ECM environments. Tissue-specific ECM composition or inflammatory triggers linked to infection may drive additional motility patterns, such as Levy walks described for CD8^+^ T lymphocytes in *T. gondii* infected brain tissues^13^. Additionally, tissue architecture is sought to provide favored routes and to impose physical constraints that dictate lymphocyte deformation and motility^41^. Independently from the complexity of *in vivo* environments, our data point to the fact that the random motility behaviors of lymphocytes are intrinsic properties of those cells and that they adapt their random motility to ECM composition and possibly related environmental cues, such as matrix fiber spacing and ECM gradients. The plasticity of lymphocyte motility is probably tuned to ensure optimal activation and effector function. Furthermore, we postulate that lymphocytes belonging to distinct subsets and maturation stages may tune differently their motility behavior, because of specific interactions with ECM components.

Our study further highlights that at high cell densities, B lymphocytes alternate between individual and collective behaviors. Importantly, cell-matrix interaction appears to affect cell-cell interaction dynamics, which control the assembly into clusters. Such interplay between cell-matrix and cell-cell interactions has previously been described for neural crest cells^42^ and *Dictyostelium discoideum*^26^. The low adherence condition (Collagen IV) favors aggregation of cells into radial clusters via their uropods in a way reminiscent of previous observations^27^. On the other hand, B lymphocytes over fibronectin fail to engage in stable cell-cell contacts. Our data point to a contact inhibition of locomotion mechanism, as described in other cells^42–44^. Our data show that the nature of the matrix, rather than its local concentration conditions specific cell behaviors. This calls for further exploration of whether the physical properties of the matrices, rather than their biochemical nature or the specificities of the corresponding adhesion receptors might explain the distinct cell behaviors. Interestingly, cell-matrix and cell-cell interactions are associated to seemingly distinct actin cytoskeleton modules. We therefore speculate that the interplay between cell-matrix and cell-cell interactions is controlled via a crosstalk between those 2 actin-rich areas. Along this concept, the different adhesive areas over the 2 matrices are associated to distinct protrusive activities impacting on the establishment of cell-cell contacts. This is reinforced by the finding that Arp2/3 is involved in cell-cell adhesion stabilization, rather than in matrix interaction. Given its role in protrusion stability and LFA-1 activation^45,46^, Arp2/3 could prime lamellipodia-like extensions that favor extensive contacts between cells, further eliciting LFA-1 driven homotypic adhesions as described for leukemic cells^7^. This is in agreement with the recent finding that the lamellipodium of lymphocytes is dispensable for cell traction during motility, but holds an exploratory role^47^.

The concept that hematopoietic cells are solitary cells and migrate independently from each other has recently been challenged by reports showing that both myeloid and lymphoid cells can switch from single cell to group behaviors^5,48,49^. Our study indicates that B lymphocyte clusters form cellular hubs that could combine the necessity for B lymphocytes to cooperate during activation and/or function, with the necessity to scan actively tissue surfaces and to recirculate. Indeed the equilibrium between clusters and individual cells is highly dynamical since groups are undergoing active fusion and fission events allowing most cells to temporally contribute to clusters. Like for the grazing dynamics in flocks of sheep^50^, B lymphocyte populations appear to balance the conflicting needs of spreading and gathering. Whereas Merino sheep alternate between group spreading and aggregation phases, B lymphocytes establish a rather stable equilibrium between compact groups and freely moving cells at the population level. Those properties might contribute to the ability of B lymphocytes to both circulate as isolated cells and to assemble into dense follicles in lymphoid organs. Additionally, the specific nature of the reticular ECM fibers composing the scaffold of the B cell follicles is expected to play a key role in the compartmentalization and dynamic behaviors of those cells^40^. A further challenge will be to investigate how the multiple 3D cell-matrix adhesions and inter-cellular interactions are regulating the multi-cellular dynamics of B cell follicles.

Our study reveals the role of cell matrix interaction on the onset of B lymphocyte collective motility. Over fibronectin, B cell clusters display reduced basal motility and chemotaxis as a function of size, pointing to the lack of coordination among the cells composing the clusters. In contrast, over collagen IV, B cell clusters display increased diffusion and chemotaxis as compared to individual cells. Both behaviors are qualitatively consistent with the Einstein relation for diffusing particles submitted to an external field. Moreover, the onset of collective chemotaxis is not the consequence of the preferential aggregation of the most motile B lymphocytes. This instead points to a cell-cell coordination mechanism. Importantly, we show that the role of extracellular matrix on the onset of collective chemotaxis applies to primary B cells isolated from human peripheral blood. In the context of tumoral lymphocytes displaying a similar collective chemotaxis, we previously established that cell coordination was ensured by the turn-over of leader cells expressing high levels of chemokine receptor and protruding toward the chemokine source^7^. Such coordination might apply to the non-tumoral B lymphocytes studied here. In light of our studies, a possible explanation for B lymphocyte coordination over collagen IV would be that cell clusters are pulled by exchanging leader cells that transiently engage in strong attachment to the matrix. Concomitantly, pulling of the cell clusters by such leader cells would be favored by the stability of cell-cell interactions. On the other hand, under conditions favoring strong attachment of most cells, the loose cell-cell contacts are generating noise that counteracts diffusion and chemotaxis. Such noise might be explained by cell-cell frictions, contact-inhibition of locomotion, or cell-cell interactions weakening cell-matrix interaction. The importance of cell-cell interactions during collective chemotaxis over collagen IV is highlighted by our experiments with the ROCK1 inhibitor. The fact that clusters display increased chemotactic speed upon ROCK1 inhibition suggests that myosin contraction in cell clusters limits cluster compactness and onset of collective chemotaxis. This hypothesis is in line with results obtained in carcinoma cells, in which the DDR1-driven down-modulation of myosin contraction in the core of clusters is required for collective migration^51^. It is currently unclear whether collective chemotaxis of lymphoid cells might occur *in vivo* because of space constraints. We favor the view that lymphocytes might exploit collective properties to assemble in areas of high chemokine concentration, such as in the context of B cell follicles.

In conclusion, our study reveals how B lymphocytes integrate cell-matrix adhesion and intercellular interactions to generate a vast array of motility behaviors at the population level. In particular, our work unveils the following rules pertaining to B lymphocyte motility: i) B lymphocytes are uniformly endowed with a local scanning activity, independently from matrix interaction; ii) space exploration by B lymphocyte populations relies on a wide distribution of individual cell diffusive abilities that is additionally tuned by matrix interaction; iii) transition from individual to schooling behaviors is highly dependent on matrix interaction. These rules provide a novel framework to explore how the complexity of physiological tissue environments or the activation status of lymphocytes are tuning their motility patterns.

## Methods

### Cell culture, transduction and treatments

EBV-transformed B cells (JY line) were cultured in RPMI 1640 GlutaMax, supplemented with 10% Fetal Calf Serum, minimum essential amino acids, HEPES, sodium pyruvate and Streptomycin/penicillin solution (Invitrogen) Cells were routinely screened for mycoplasm contamination using the MycoAlert mycoplasma detection kit (Lonza). For the stable expression of GFP, LifeAct-GFP, LifeAct-RFP, 1 × 10^5^ JY cells were transduced with a lentiviral vector encoding LifeAct-TagGFP2 or LifeAct-TagRFP (Ibidi) over 6 h in RPMI-1640 containing 5% FCS and polybrene (4 μg ml^−1^). Transfected cells were selected by culturing them in the presence of 2 μg ml^−1^ puromycin for 7 days, added 24 h after transfection. Bright GFP-positive cells were further selected by flow cytometry sorting. Where indicated, cells were treated with Y27632 (Abcam) at 10 μM, CK 869 (Sigma-Aldrich) at 50 μM or the equivalent concentration of DMSO (10^−3^). No treatment toxicity was detected in a window of 16 h. Ibitreat^TM^ (Ibidi) plastic plates were coated with 1,5 μg ml^−1^ collagen type IV from human placenta or 10 μg ml^−1^ fibronectin from human plasma (both from Sigma-Aldrich) diluted on PBS. Matrices were incubated ON at 4 °C and 1 h at 37 °C before washing with medium and cell seeding.

### Primary B cell purification and culture from peripheral blood

Buffy coats from healthy donors were obtained through the Etablissement Français du Sang (EFS Midi-Pyrénées, Purpan University Hospital, Toulouse, France) and processed following standard ethical procedures (Helsinki protocol), after obtaining written informed consent from each donor and approval by the local ethics committee (Comité de Protection des Personnes Sud-Ouest et Outremer II). B cells were isolated from buffy coats via separation by Ficoll centrifugation and a negative selection kit (EasySep Human B Cell Enrichment Kit; Stem Cell Technologies). Purified B cells were cultured at 5 × 10^6^ cells ml^−1^ in RPMI 1640 GlutaMax, supplemented with 10% Fetal Calf Serum and stimulated for 48 h with the 5 μM CpG ODN 2006 (InvivoGen) for 48 h before performing the experiments.

### Expression of integrins

JY cells or PBMCs were washed and blocked for 20 min with PBS, 20% human serum at 4 °C. Then, cells were stained with antibodies against CD19, CD49a, CD49b or CD49d (Biolegend) at 10 µg/ml for 60 min at 4 °C. Stained cells were washed several times and analyzed using a Miltenyi MacsQuant VYB flow cytometer. Using the FlowJo software, B cells were selected by gating on the CD19 positive cells and analyzed for their expression of the CD49a, CD49b and CD49d integrin alpha-chains.

### Recording and quantification of B lymphocyte motility

JY cells were washed, resuspended in fresh medium and seeded (5 × 10^3^ cells in 120 µl) onto a collagen IV or fibronectin coated channel from a µ-Slide VI 0,4 (Ibidi) to reach a cell density of 50 cells mm^−2^. After 2 h incubation at 37 °C to allow cell attachment, cells were recorded for 12 h at a rate of 1 image each 5 s on an Eclipse TE2000-E fully-motorized inverted microscope (Nikon) and a 10x/0.45 NA objective. The microscope was equipped with an incubation chamber (OKOlab) for temperature and CO_2_ control. Images were acquired using a Evolve 512 emCCD camera (Photometrics) and the Metamorph software (Molecular Devices). Image processing was performed with a program written in C+ +, using the open source OpenCV library. To identify the interactions, the first step consisted in detecting and outlining the individual cells and groups. The images were binarized using Otsu method and a morphological closure was applied to reduce noise. Edge detection was implemented with the Suzuki method and the further blob labeling provided a map at any time point (t). Secondly, fusion and scission dynamics were analyzed over time by matching blobs at time t-1 with the ones found at time t. Briefly, distance, size and cell density index combination provided a score for the best blob matching. In case of several blobs with the same match, a fusion or scission event was considered. In case of a missing match, the cell (or cluster) passed to “standby mode” being considered as definitively lost if it did not reappear for 10 frames (50 sec). This method helped us to overcome the problem of over-labeling of “intermittent” cells/clusters due to faint contrasted contours. The proportion of lost cells or clusters in the analyzed video-recordings ranged between 0.44% and 1.33%, depending on ECM coating and cell density. The proportion of gaps in the analyzed tracks ranged between 1.94% and 3.10%, depending on ECM coating and cell density.

### B lymphocyte clustering by fixed imaging

JY cells were seeded at 1.2 × 10^4^ cells per well in an angiogenesis µ-plate (Ibidi) containing either uncoated wells or wells precoated with collagen IV (1,5 or 10 µg/ml) or fibronectin (1,5 or 10 µg/ml). The plate was placed under a Zeiss apotome microscope equipped with a chamber (37 °C, 5% CO_2_) and imaged in a bright field mode at 0, 1, 2, 4 and 6 h, using a 10x/0.45 NA objective and an Axiocam HRm Rev.3 camera. The image treatment from previous experiments was applied to recognize the perimeter of all cell groups present at each time point. Then, the mean size of individual cells was calculated for each experimental condition and each group area was referred to that value in order to estimate the number of cells composing it. We further checked that the area of clusters remained proportional to the number of cells composing them by staining and counting individual cell nuclei.

### B lymphocyte clustering by video recording

JY cells were seeded over the different matrices at 10^3^ cells mm^−2^. Cells were imaged for 12 h with the same settings as in the above section, except that single frames at a few time intervals were replaced by continuous imaging at a rate of 1 frame each 30 s. Where indicated, cells under were treated for 2 h before seeding with either Y27 or CK869. These drugs were kept in the cell medium during the entire recording. The same image analysis tools as above were applied to detect, count and estimate the size of cells and cell groups.

### Cluster fusion and fission

Data corresponding to the area and position of cells and cell groups recognized along the video-recordings were computed. On that basis, fusion events were identified as the disappearance of two groups in a frame linked to the appearance of a group of a larger area at the same location in the successive frame. Fission events were defined as the disappearance of a group in a frame linked to the appearance of two smaller groups at the same location in the successive frame. Both fusion and fission events were classified by size category (n) and the crossed probability of fusion and fission for the different size established as P fusion (n1, n2) = [Number of fusions n1 + n2 per min *(Mean number of clusters from all size categories during the 6 h recording)/(Mean number of clusters from n1 size category along the 6 h *Mean number of clusters from n2 size category along the 6 h)] and P fission (n→n1 + n2, with n1 ≤ n2) = (Number of fissions n→n1 + n2 per min/Mean number of clusters during the 6 h recording).

### Tracking of individual cell association to clusters

JY cells were prepared at a 1/40 GFP positive cell proportion, seeded over collagen IV or fibronectin as before and incubated for 6 h at 37 °C. The bright field and GFP channels were imaged for 6 h each 30 s on an Apotome microscope (Zeiss) and a 10x/0.45 NA objective, as described in the above section. The Image J software was used for video quantification. Briefly, the bright field channel was used to generate a binary image. Then specific masks for individual cells, groups from 2 to 5 cells and groups composed of 6 cells or more were applied and a specific signal intensity was associated to each mask. The three masks were added to recompose the original binary image, this time with an intensity code to distinguish between the three size classes. The GFP channel was used to generate a mask that identified each cell for the full video. Combination of the recomposed bright field and GFP masks and analysis with the TrackMate Image J plugin^52^ provided a tool to follow motility and clustering simultaneously. Cell speed and relative time in each size category were extracted. Speed values obtained from individual tracks shorter than 5 min were discarded. Correlation between speed and time of cluster residence/isolation represented for each cell by using GraphPad Prism.

### Combinatorial TIRF, IRM, and widefield analysis of actin dynamics

JY cells expressing LifeAct-GFP were prepared and seeded on collagen IV or fibronectin at low or high cell density as in previous experiments. After 2 h at 37 °C for cell adhesion and, in case, treatment action, cells were placed on a Nikon inverted spinning disk confocal microscope equipped with a back-thinned charge-coupled device camera (Evolve; Photometrics, Tucson, AZ, 512 × 512 pixels), equipped with a temperature and CO_2_ controlled chamber and imaged with an oil immersion 100x/1.49 NA objective. 491 nm laser was used to obtain TIRF and widefield at the cell-matrix contact focus, while IRM image was obtained by using 561 nm laser. Added to that, widefield 491 nm signal and brightfield were obtained at cell midbody focus, where cell protrusion where extended. Fixed images or live videos with a 10 s temporal resolution were taken. Relative cell attachment area was calculated by dividing TIRF LifeAct-GFP area by widefield area for each cell. Cell shape volatility was calculated using widefield LifeAct signal as reference for cell shape. Aspect ratio was calculated by dividing the major axis by the minor axis of each cell at each frame using Image J. Volatility was calculated as the standard deviation of the aspect ratio changes over consecutive frames.

### Primary B cell attachment, motility and dynamics

Primary B cells were seeded over collagen IV or fibronectin and cultured for 2 h. Cells were imaged on a Nikon inverted spinning disk confocal microscope with a 100X objective for brightfield and IR illumination. Fixed images from random fields with at least 3 cells were taken and cells showing a contact area with the matrix (altering the IR signal pattern) were quantified. For motility studies, 10^5^ cells were seeded at 10^3^ cells mm^−2^ and imaged for 16 h at a frame rate of 1 image each 30 s on a Zeiss apotome microscope equipped with a chamber (37 °C, 5% CO_2_) and imaged in a bright field mode with a 10x/0.45 NA objective. For motility quantification, images were treated using Image J to select individual cells by their size and contrast to background and tracks over 20 min appearing after 3 h of recording were automatically registered and quantified using Trackmate. For population dynamics, individual cells, small (2 to 5 cells) and large clusters (6 or more cells) were detected and their covered surface quantified with Image J. The relative proportion of each size category was quantified and plotted with Graphpad Prism.

### Chemotaxis assays and 2D migration

JY cells or primary B cells (10^5^ in 6 µl of culture medium) were loaded into the central transversal channel of Ibitreat 3D chemotaxis µ-slides (Ibidi) coated with collagen IV or fibronectin and incubated at 37 °C for 30 min to allow cell attachment. CCL19 0–500 ng ml^−1^ gradient was created following the manufacturer’s instructions. To verify the linearity of the obtained gradients, control slides were prepared using a 10% dextran-FITC solution. Analysis of fluorescence intensity profiles showed that linear gradients were established within 3 h and were stable over at least 24 h. Cell migration was recorded after 5 h by taking 1 image per 30 s for 14 h by an Apotome microscope (Zeiss) and a 5x/0.15 NA objective in temperature and CO_2_ controlled conditions. Obtained images were treated and binarized using Image J to create masks corresponding to individual cells, small (2 to 5 cells) and large (6 or more cells) clusters. For primary B cells, the relative paucity of clusters led us to consider only one cluster category. Migration tracks of each category were obtained using the TrackMate plugin of the FIJI software. Only tracks lasting more than 20 min were considered. Chemotaxis plots and migration parameters (FMI-Y and total speed) were obtained with the Chemotaxis and Migration tool from Ibidi. Chemotactic speed was calculated by multiplying mean speed by FMI-Y for each cell or cluster.

### Availability of data and material

The datasets used and analyzed during the current study are available from the corresponding author on reasonable request.

### Ethics approval and consent to participate

Primary B cells were purified from blood samples collected from healthy adult donors. Samples were obtained as buffy coats generated upon altruistic donation of blood at the Etablissement Français du Sang (EFS Midi-Pyrénées, Purpan University Hospital, Toulouse, France). All samples were collected after signing an informed consent, following standard ethical procedures (Helsinki protocol) and with the approval by the local ethics committee (Comité de Protection des Personnes Sud-Ouest et Outremer II).

## Electronic supplementary material

Supplementary Information

Video legends

Video 1

Video 2

Video 3

Video 4

Video 5

Video 6

Video 7

Video 8
