# Supplementary material for: Switching between individual and collective motility in B lymphocytes is controlled by cell-matrix adhesion and inter-cellular interactions
Source: Sci Rep. 2018 Apr 11;8:5800. doi: 10.1038/s41598-018-24222-4 (PMC5895587; doi:10.1038/s41598-018-24222-4)
Supplement: Supplementary file 1 — Supplementary Information [file 41598_2018_24222_MOESM1_ESM.pdf]

Switching between individual and collective motility in B lymphocytes is controlled by cell-matrix adhesion and inter-cellular interactions

Javier Rey-Barroso, Daniel S. Calovi, Maud Combe, Yolla German, Mathieu Moreau, Astrid Canivet, Xiaobo Wang, Clément Sire, Guy Theraulaz and Loïc Dupré

SUPPLEMENTARY INFORMATION

→ Supplementary Figures S1 to S6

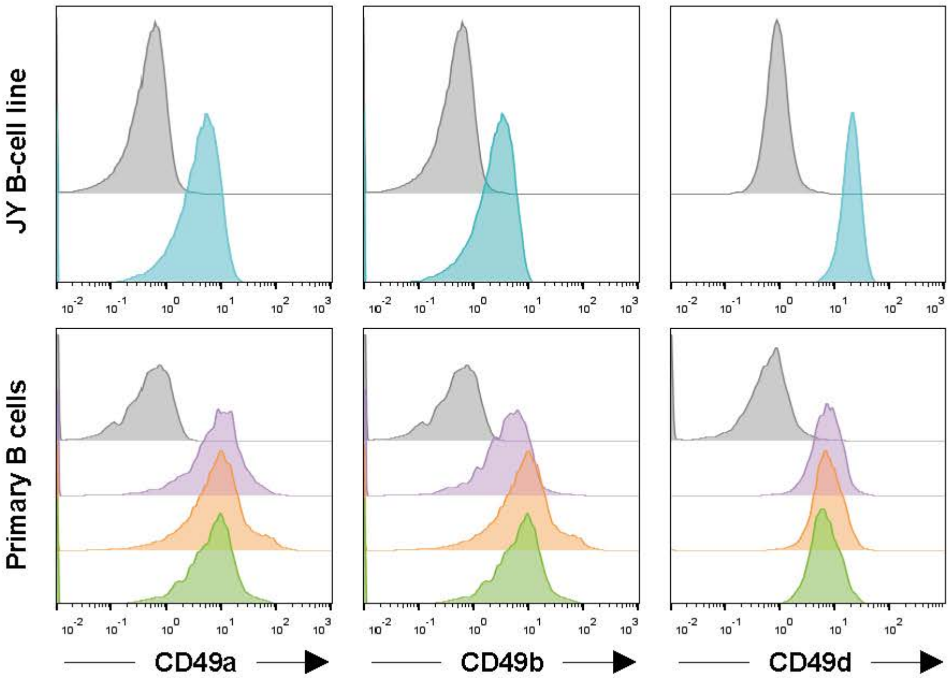

Supplementary Figure S1. B cells from the JY line and primary B cells express comparable and homogeneous levels of integrins.

Expression levels of the integrin alpha chains CD49a, CD49b and CD49d on JY cells or primary B cells from the peripheral blood of three healthy donors were analyzed by flow cytometry and represented as histograms. Negative controls are plotted in grey for comparison.

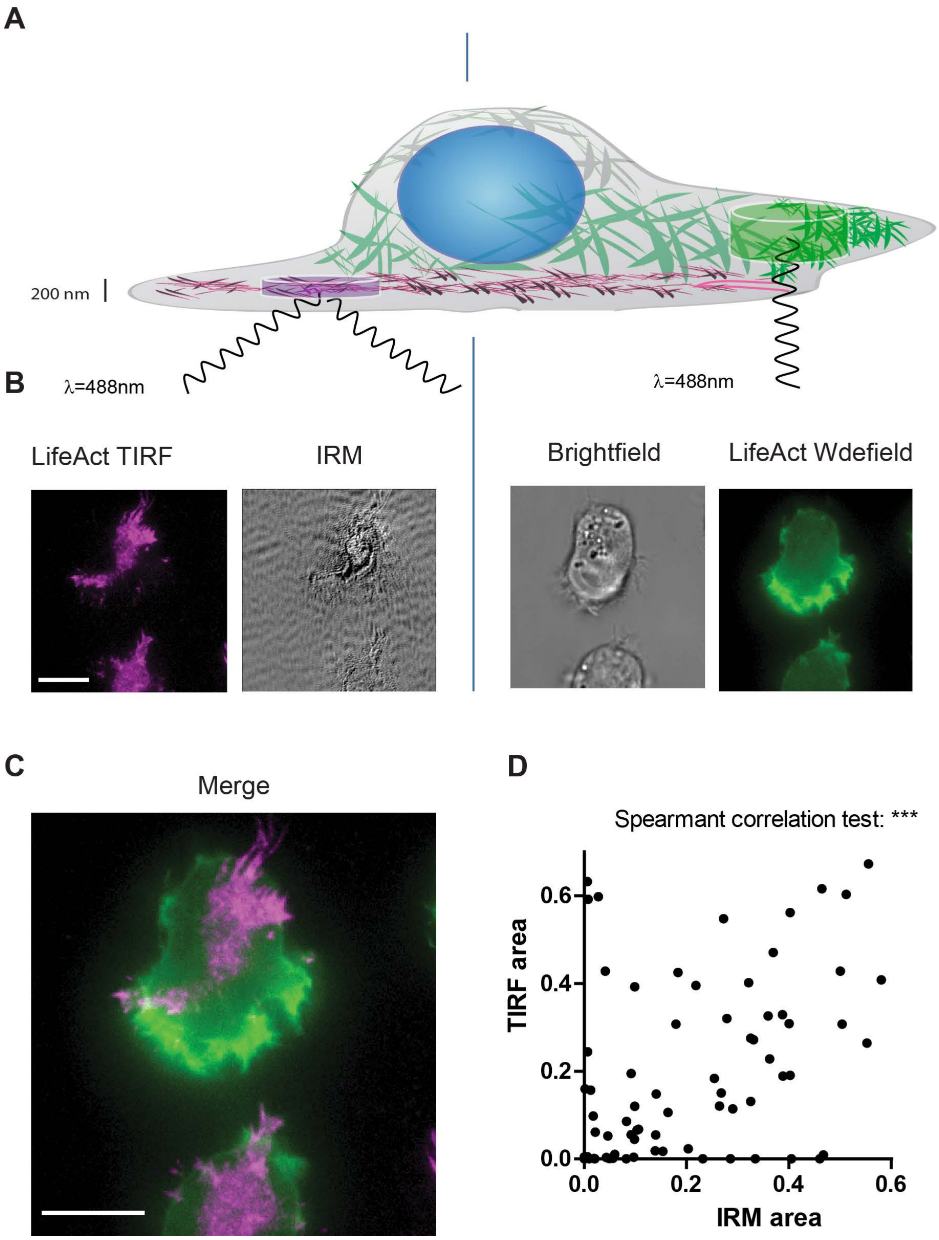

Supplementary Figure S2. Combinatorial microscopy reveals adhesive and protrusive actin modules.

(A) Schematic representation of a LifeAct-GFP expressing cell examined by a real-time microscopy approach combining oblique illumination for TIRF (LifeAct-GFP, represented in magenta) and IRM and widefield illumination (brightfield and LifeAct-GFP, represented in green). **(B)** Representative LifeAct-GFP expressing JY cell on fibronectin imaged through the combinatorial microscopy approach. Scale bar: 10 µm. **(C)** Overlay of the LifeAct-GFP images obtained via widefield and TIRF illumination modes. Scale bar: 10 µm. **(D)** Analysis of the correlation between the LifeAct-GFP TIRF and IRM areas in LifeAct-GFP expressing JY cells (n=73 cells).

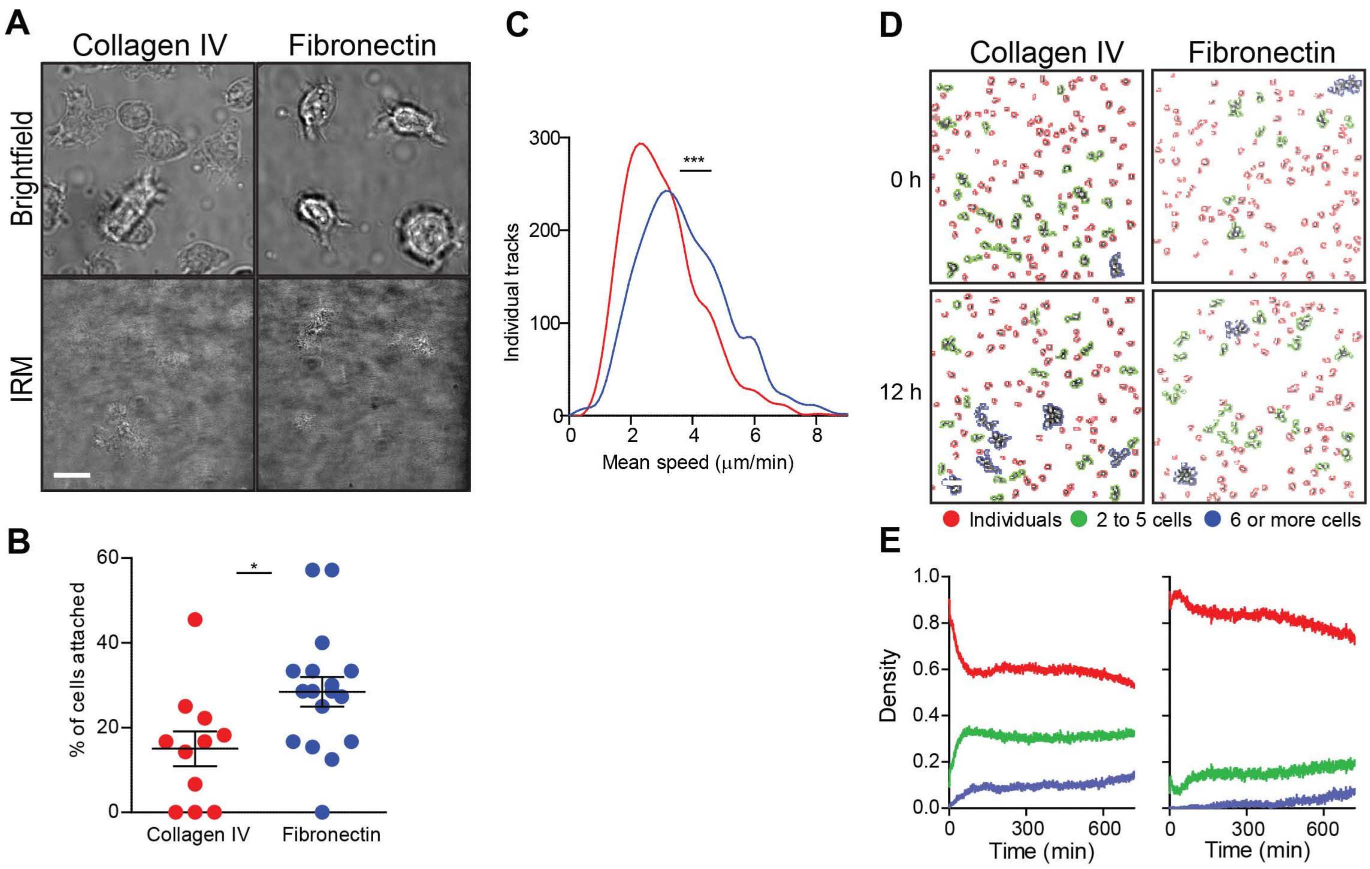

Supplementary Figure S3. Matrix interaction defines primary B cell motility and self-assembly into clusters.

(A) Human primary B cells from peripheral blood stimulated 48 h with CpG2006 were seeded over collagen IV of fibronectin. After 4 h cells were imaged by brightfield and IRM to detect attachment state. **(B)** Quantification of cell attachment to matrix by calculation of percentage of cells showing contact on IRM divided by total number of cells in the image (n=107 from 11 images for collagen IV and 137 from 17 images on fibronectin). Bars show mean and SEM. * $p<0.05$, Student-t test. **(C)** Primary B cells seeded over collagen IV or fibronectin were imaged for 12 h and tracks over 10 min recorded and computed. Track mean speed (time lag 30 s) distribution was represented (n=1694 and 1687 cells, from two independent experiments) *** $p<0.001$, Student-t test. **(D)** Primary B cells population dynamics over 12 h after seeding over collagen IV or fibronectin at high density were recorded, and individuals, small (2 to 5 cells) and large (6 or more) groups were detected and color coded. Images show a representative population distribution at time 0 and 12 h. **(E)** Evolution of the relative presence of individual and 2 to 5 cell clusters and 6 or more cell clusters (mean from n=2 videos).

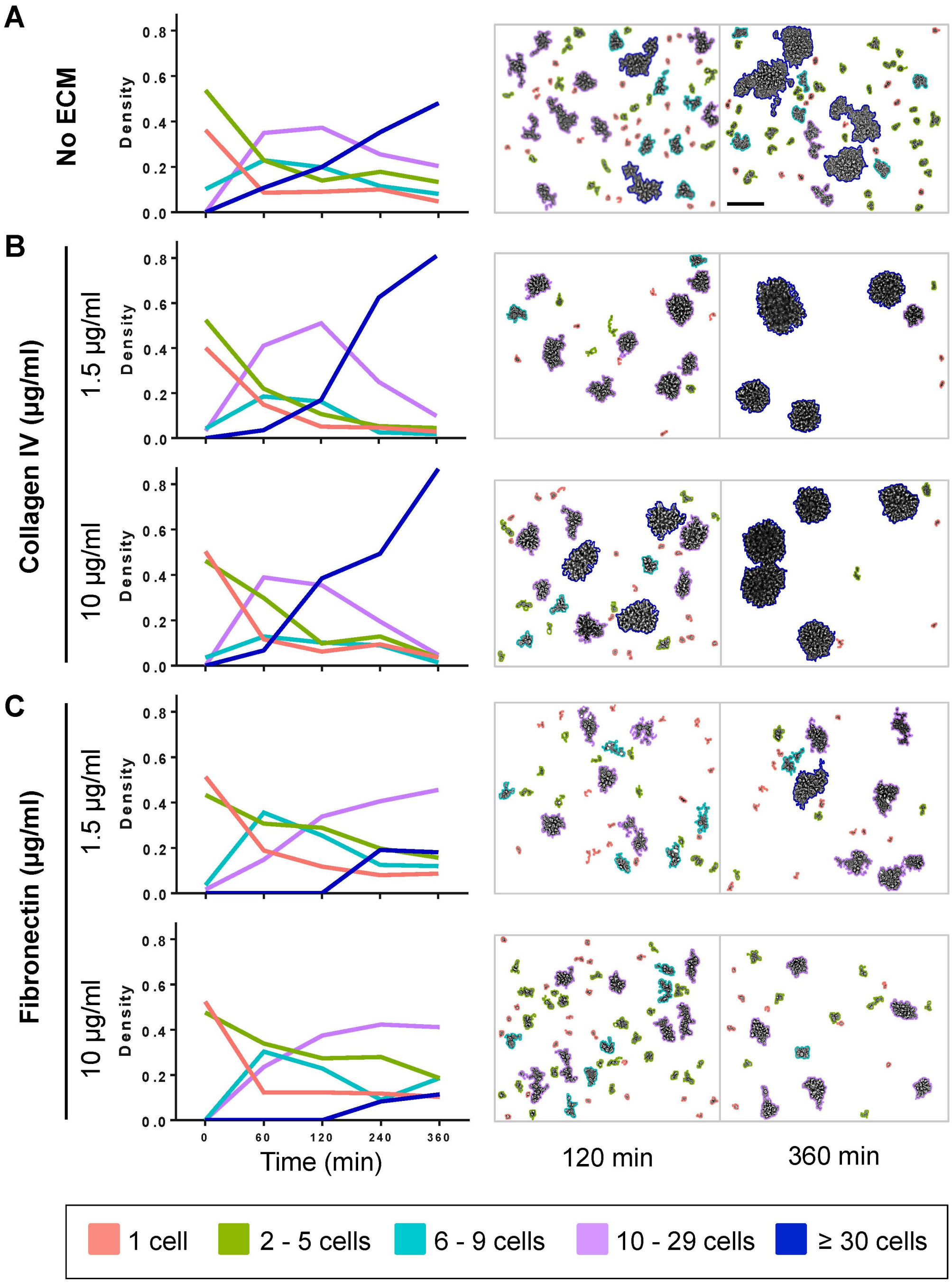

Supplementary Figure S4. Effect of extracellular matrix composition and concentration on lymphocyte assembly into clusters.

JY cells were seeded over **(A)** plastic, **(B)** collagen IV (1,5 or 10 µg/ml) or **(C)** fibronectin (1,5 or 10 µg/ml) and imaged at different time points. Cluster area was recognized and normalized by mean cell size to generate 5 subcategories, as indicated. Relative area covered by each subcategory was calculated and its temporal evolution represented. Representative pictures with colored contours of clusters of each category are shown for each condition at 120 or 360 min. Scale bar, 100 µm. 3 to 6 fields were collected and integrated in the analysis for each condition.

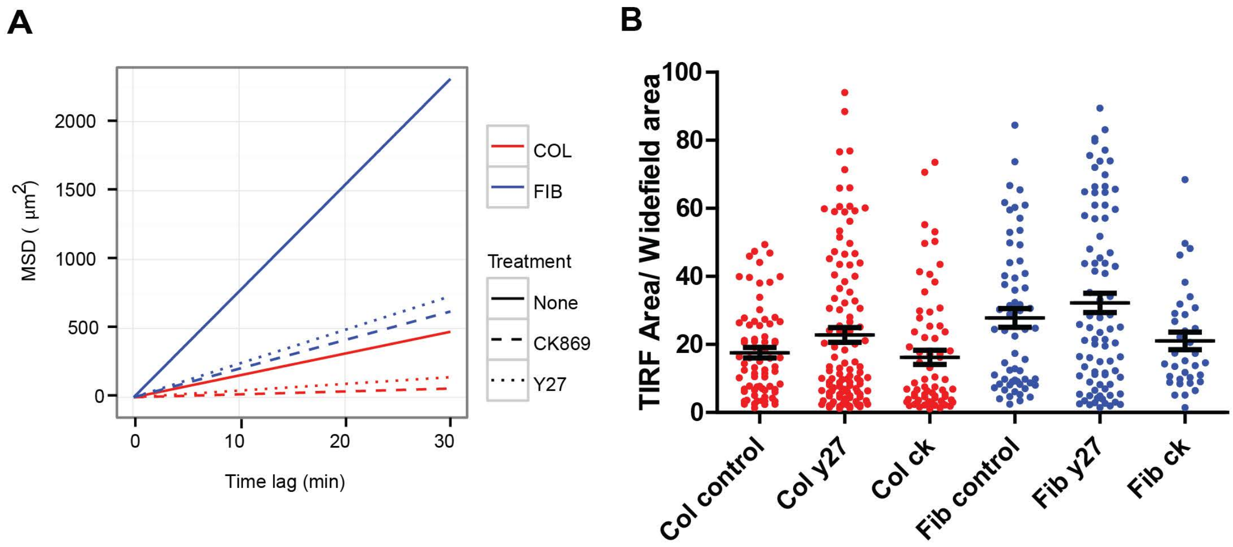

C

		Diffusion coefficient (μm2/min)	Mean Speed (time lag 5 sec) mm/min	Mean displacement (time lag 5 min) mm/min	Motile efficiency (Mean displacement/mean speed)
COL	Control	3,5	11,05	1,67	0,15
	CK	0,6	7,7	0,69	0,09
	Y27	1,3	6,92	1,02	0,15
FIB	Control	18,5	10,9	3,84	0,35
	CK	5,2	8,5	2,03	0,24
	Y27	5,95	6,95	2,18	0,31

Supplementary Figure S5. Roles of actin branching and actomyosin contraction in lymphocyte attachment and motility.

(A) Tracks from Y27632 or CK869 or non-treated JY individuals seeded over collagen IV or fibronectin were obtained from high cell density videos. All tracks over 1 min were computed for each condition and Mean Square Displacement evolution quantified, fitted and represented. **(B)** Y27632 or CK869 or non treated JY cells were seeded over collagen IV or fibronectin and imaged on TIRF and widefield after 3h. Respective cell areas were calculated and a ratio between them was established and represented. Bars show mean and SEM (n=79, 119, 76, 65, 89 and 38 cells). **(C)** Tracks from Y27632 or CK869 or non-treated JY individuals seeded over collagen IV or fibronectin were analyzed to reveal their mean speed. Then diffusion coefficient and mean displacement per min (time lag of 5 min) were extracted from MSD calculation and the motile efficiency was calculated as the ratio between the mean displacement and the mean speed. Results were represented as a table, color-coded respect to their value.

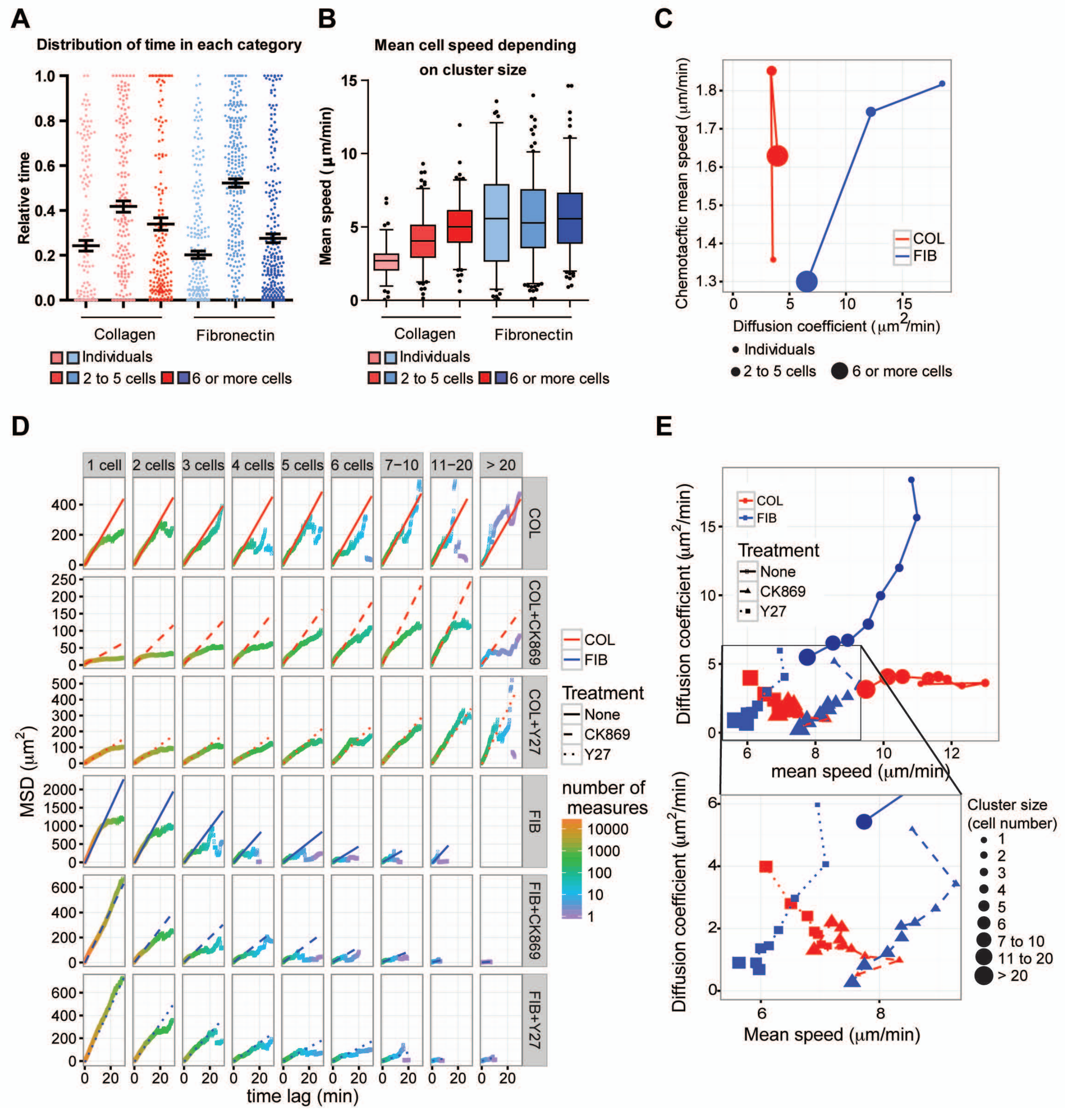

Supplementary Figure S6. Effect of matrix composition on lymphocytes propensity to assemble into clusters or remain isolated, and on their speed during each of these phases.

(A) JY-GFP cells diluted 1/40 into unstained JY were seeded over collagen IV or fibronectin and recorded for 12 h. GFP cells relative time as individual or part of 2 to 5 or 6 or more cells clusters was quantified and plotted (n=178 and 250, from three independent experiments). Bars show mean and SEM. **(B)** Mean speed of JY-GFP cells (considering tracks over 10min) when being isolated or part of clusters was quantified and plotted for each condition (n=90, 144, 124, 132, 228 and 177 from a representative experiment). Box and whiskers plot, with a 5 to 95% data range and values out of the range as single points. **(C)** Cluster size categories in absence of gradient were compiled into the three categories made for gradient experiments and their diffusion coefficient calculated. Plot shows comparison of chemotactic mean speed and diffusion coefficient in the same three categories. **(D)** MSD of tracks from Y27, CK869 or non-treated JY cell clusters of growing size were obtained from high cell density videos over collagen IV or fibronectin and shown in red or blue, respectively. Color code lines show original values and number of tracks available for each time length. **(E)** Diffusion coefficients of each cluster category were extracted from previous graph and plotted respect to their mean speed (time lag 5 s).
