# Supplementary material for: Switching between individual and collective motility in B lymphocytes is controlled by cell-matrix adhesion and inter-cellular interactions
Source: Sci Rep. 2018 Apr 11;8:5800. doi: 10.1038/s41598-018-24222-4 (PMC5895587; doi:10.1038/s41598-018-24222-4)
Supplement: Supplementary file 2 — Video legends [file 41598_2018_24222_MOESM2_ESM.doc]

**Switching between individual and collective motility in B lymphocytes is controlled by cell-matrix adhesion and inter-cellular interactions**

Javier Rey-Barroso, Daniel S. Calovi, Maud Combe, Yolla German, Mathieu Moreau,

Astrid Canivet, Xiaobo Wang, Clément Sire, Guy Theraulaz and Loïc Dupré

SUPPLEMENTARY INFORMATION

🡪 Legends of supplementary videos

**Supplementary Video S1. JY cell shape remodeling and motility over collagen IV and fibronectin.**

The composed movie shows examples of:

- LifeAct-GFP JY cells seeded over collagen IV or fibronectin and imaged by combinatorial microscopy, used for Figure 1C quantifications.

- JY cells seeded over collagen IV or fibronectin and imaged by brightfield microscopy, used for Figure 1D-F, and Figure 2A-C quantifications.

**Supplementary Video S2. JY cells displaying either poor, dynamic and sustained attachment to matrix.**

The composed movie shows representative examples of LifeAct-GFP JY cells seeded over fibronectin and imaged by combinatorial microscopy, used for Figure 2D quantifications.

**Supplementary Video S3. JY cell aggregation process.**

The composed movie shows examples of:

- Aggregation events and cluster identification after collision correction, used for Figures 3A-D, 4A-C, 5A-C and 6E

- JY cells or primary B cells seeded at high cell density over collagen IV or fibronectin and imaged by brightfield microscopy for 12h, used for Figure 3A-D, 5A-C, 6E and Supplementary Figure S2 quantifications.

**Supplementary Video S4. LifeAct-GFP JY cell-cell interaction dynamics over collagen IV and fibronectin.**

The composed movie shows representative examples of LifeAct-GFP JY cells seeded over collagen IV or fibronectin and imaged by combinatorial microscopy, used for Figure 3E.

**Supplementary Video S5. Effect of Y27 and CK869 on JY cell-cell interaction dynamics.**

The composed movie shows representative examples of Y27 or CK869 treated LifeAct-GFP JY cells seeded over collagen IV or fibronectin and imaged by combinatorial microscopy, used for Figure 4D quantifications.

**Supplementary Video S6. Correlation between JY cell motility and propensity to associate to clusters.**

The composed movie shows representative examples of GFP JY mixed with non-marked JY cells seeded over collagen IV or fibronectin, imaged by widefield microscopy, and color-coded by their association to clusters of different size, used for Figure 5D and Supplementary Figure S4 quantifications.

**Supplementary Video S7. Individual and collective JY and primary B cell 2D chemotaxis.**

The composed movie shows representative examples of JY cells or primary B cells seeded at high cell density over collagen IV or fibronectin, exposed to a 0-500 ng/ml CCL19 gradient, imaged by brightfield microscopy and tracks analyzed depending on cluster size, used for Figure 6A-D quantifications.

**Supplementary Video S8. Effect of Y27 and CK869 on individual and collective B cell chemotaxis.**

The composed movie shows representative examples of Y27 or CK869 treated JY cells seeded at high cell density over collagen IV or fibronectin, exposed to a 0-500 ng/ml CCL19 gradient, imaged by brightfield microscopy and tracks analyzed depending on cluster size, used for Figure 6F quantifications.
